# Imperatorin–pharmacological meaning and analytical clues: profound investigation

**DOI:** 10.1007/s11101-016-9456-2

**Published:** 2016-02-20

**Authors:** Ewelina Kozioł, Krystyna Skalicka-Woźniak

**Affiliations:** Department of Pharmacognosy with Medicinal Plant Unit, Medical University of Lublin, 1 Chodzki Str., 20-093 Lublin, Poland

**Keywords:** Imperatorin, Furanocoumarin, Psoralen derivatives

## Abstract

Imperatorin, a furanocoumarin derivative, has many documented pharmacological properties which make it a candidate for possible drug development. In this review, the activity on the central nervous system, the anticancer and antiviral properties and the influence on the cardiovascular system are described. The aim of this review is also to present an overview of the techniques used for the analysis, isolation, and separation of imperatorin from plant material from the practical perspective.

## Introduction

**Fig. 1:**
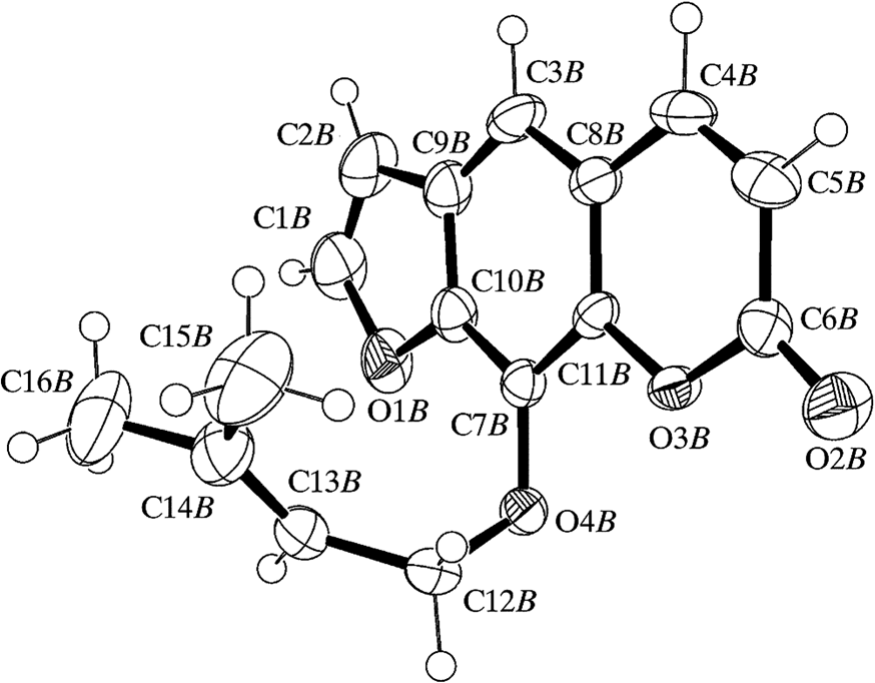
The molecular structure of imperatorin (Cox et al. 2003)

Imperatorin, known as 9-[(3-methyl-2-buten-1-yl)oxy]-7*H*-furo[3,2-*g*]chromen-7-one or 8-(1,1-dimethylallyloxy)-psoralen, is a plant secondary metabolite belonging to the coumarins—specifically the furanocoumarins. It has a molecular weight of 270.27996 amu and corresponding to the molecular formula C_16_H_14_O_4_. Physically, the pure substance presents as white, long fine needles or crystals, m.pt. 101 °C. Imperatorin is insoluble in water and is easily soluble in nonpolar solvents (Cox et al. 2003). The molecular structure of imperatorin showing the atomic arrangement is presented on Fig. 1.

**Fig. 2:**
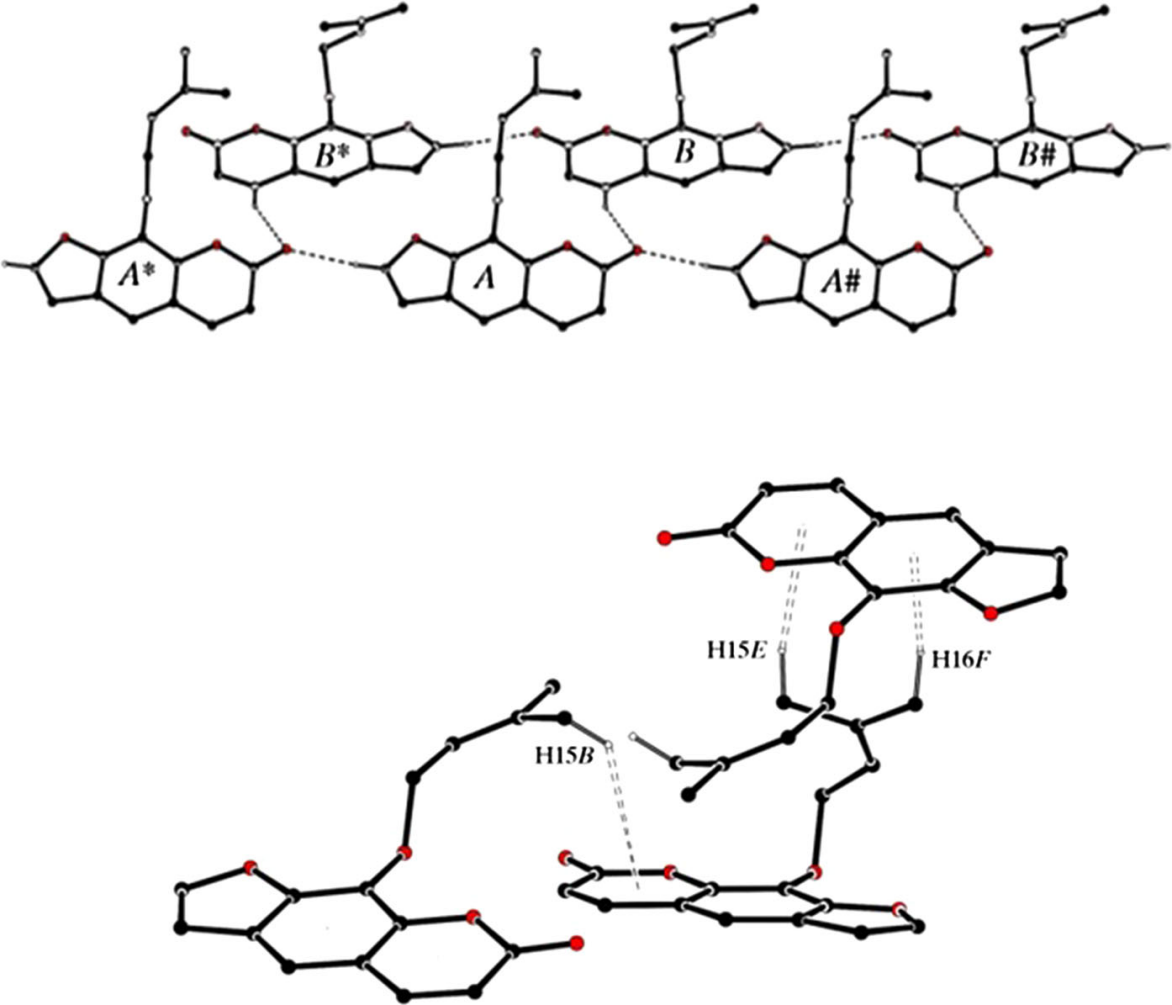
Intra- and intermolecular interactions between molecules of imperatorin in the crystal lattice (Cox et al. 2003)

The pure compound forms crystals [*a* = 11.0551 (4) Å, *b* = 11.7236 (4) Å, *c* = 11.8718 (5) Å, *α* = 64.1855 (18) Å, *β* = 89.6613 (16) Å, *γ* = 83.484 (2) Å] and the interactions between molecules in the crystal structure are shown on Fig. 2. The structure is stabilized by the intramolecular and intermolecular C–H–O interactions (Cox et al. 2003).

In plants, imperatorin is synthesized, as are other furanocoumarins, through the shikimic acid pathway (Bourgaud et al. 2006). Imperatorin is distributed mainly among plants from the Apiaceae and Rutaceae families, known from many traditional applications, as summarized in Table 1. Imperatorin was noted also as a constituent in many traditional medicaments, especially in Traditional Chinese Medicine (TCM). Some examples are indicated in Table 2.

**Table 1:** Plants containing imperatorin as an important constituent and their traditional use

Species	Family	Occurrence	Traditional use
*Angelica dahurica* (Fisch. ex Hoffm.) Benth, et Hook	Apiaceae	East Russia, Asia	For headache, toothache, nose congestion resulting from cold, analgesia, acne, ulcers, carbuncle, rheumatism, as sedative (Baek et al. 2000; Chen et al. 2008; Kang et al. 2008)
*Glehnia littoralis* (G.) Fr. Schmidt ex Miquel	Apiaceae	China, Japan, Canada and America	As a tonic, antiphlogistic and mucolytic medicine for the treatment of respiratory and gastrointestinal disorders, as diaphoretic, antipyretic, analgesic, antibacterial, and antifungal (Yang et al. 2010)
*Niphogeton ternata*Willd. ex Schltr.	Apiaceae	Colombia	For dysentery, colitis and rheumatism (Duan et al. 2002)
*Ostericum koreanum* (Maxim.) Kitag.	Apiaceae	South Korea	For cold, headache, neuralgia and arthritis (Lee et al. 2008)
*Peucedanum officinale* Lindl, *Peucedanum coriaceum* Reich.	Apiaceae	South and Middle Europe, Croatia	As an insect repellent (Hadaček et al. 1994)
*Peucedanum ostruthium* (L.) W. Koch	Apiaceae	Alpine region	In gastro-intestinal diseases, and also for disorders of the cardiovascular system, the respiratory tract, and to treat tiredness (Vogl et al. 2011)
*Pleurospermum rivulorum* Diels.	Apiaceae	South China	As an antipyretic, analgesic and diaphoretic (Xiao et al. 1997)
*Prangos pabularia* Lind.	Apiaceae	North India, Central Asia	As an emollient, carminative, tonic, antiflatulent, anthelmintic, antifungal, and antibacterial (Banday et al. 2013), for bleeding and to heal scars (Tada et al. 2002)
*Prangos platychlaena* Boiss	Apiaceae	Eastern Turkey	For bleeding and to heal scars (Ulubelen et al. 1995)
*Saposhnikovia divaricata* (Turcz.) Schischk.	Apiaceae	China	For pyrexia, rheumatism, headache and convulsions (Kang et al. 2008)
*Saussurea medusa* Maxim.	Compositae	Asia, Europe, North America	For anthrax, apoplexy, rheumatic arthritis and altitude sickness (Dawa et al. 2009)
*Aegle marmelos* Correa	Rutaceae	Southeast Asia	In the treatment of malaria, diabetes, dyspepsia, constipation and body heating problems (Mishra et al. 2010)
*Clausena anisata* (Willd.) Hook. f. ex Benth	Rutaceae	West Africa	As an insect repellent (Ngadjui et al. 1989)
*Clausena ansium* Lour.	Rutaceae	Southeast Asia	For coughs and colds, asthma, gastrointestinal diseases, influenza, abdominal colic pains, as an antifungal, antiproliferative, and HIV reverse transcriptase inhibitor (Maneerat et al. 2010)
*Eisenbeckia yaxhoob* Lundell	Rutaceae	Southeast Mexico	For gastrointestinal ailments (Mata et al. 1998)
*Pilocarpus goudotianus* Tul.	Rutaceae	Colombia and Venezuela	As an allelopathic agent inhibiting germination and root length of competing species such as lettuce, tomato, cucumber, and radish (Macías et al. 1993)
*Poncirus trifoliata* (L) Raf.	Rutaceae	Eastern Asia	In treating allergic diseases, as an anti-inflammatory, anti-bacterial and anti-mucin releasing (Xu et al. 2008)
*Stauranthus perforates* Liebm	Rutaceae	Mexico (Oaxaca and Yucatan), Costa Rica and Panama	As an insect repellent (Setzer et al. 2003)

**Table 2:** Examples of TCM remedies containing imperatorin

Name of the TCM	Country	Pharmacological use
Oyaksungisan	Korea	For rheumatoid arthritis, paralysis and stroke, as an anti-inflammatory and analgesic, for relaxant activity in hypertension (Weon et al. 2012)
Ru-Yi-Jin-Huang-San	China	For reducing swelling, removing poisons, and relieving pain (Lay et al. 2010)
HouxiangZhengqi liquid	China	For reducing swelling, removing poisons, relieving pain (Li et al. 2006)
Yigong	China	In vigorating vital energy, to prolong and/or irregular menstruation (Feng et al. 2008)
Qianghuo	China	As a diaphoretic, antifebrile and anodyne (Jiang et al. 2007)
*Cnidium monnieri* (L.) Cusson	China	For impotence, frigidity, skin-related diseases, as an antiallergic, antidermatophytic, antibacterial, antifungal, antiosteoporotic (Li and Chen 2005)

## Pharmacological properties of imperatorin

Many of the pharmacological activities observed in ethnomedicine became the subject of profound studies, and a series of important biological properties were described which indicated that imperatorin is an important bioactive molecule, and may be considered as a possible structure for further drug modeling and development. Aspects of these activities will be described briefly.

### Activity of imperatorin on the central nervous system (CNS)

It has been established that coumarins, particularly furanocoumarins, are a potential valuable resource for the prevention and therapy of some CNS diseases (Skalicka-Wozniak et al. 2016). The influence of imperatorin on processes of learning, its anxiolytic effect and anti-epileptic activity were described. However, few mechanisms of action were assigned to imperatorin to explain these activities.

### In vitro studies

Some of the most important receptors in the CNS are those binding gamma-aminobutyric acid (GABA), whose activation is responsible for sedation, anxiety and for anti-epileptic effects. In in vitro experiments imperatorin enhanced the GABA-induced chloride ion current (I_GABA_) through the α_1_β_2_γ_2S_ receptors. This compound potentiated I_GABA_ at 100 µmol by 50.5 ± 16.3 % and at 300 µmol by 109.8 ± 37.7 %, respectively (Zaugg et al. 2011). However, in comparison with other coumarins, such as osthol, oxypeucedanin, or phellopterin, the enhancement of the activity of GABA_A_ receptor by imperatorin was moderate. The potentiation values for the mentioned compounds, tested at 100 µmol on recombinant α_1_β_2_γ_2S_ GABA_A_ receptors expressed in *Xenopus laevis* oocytes was 124.5, 550, 56.5 and 54.1 % for osthol, oxypeucedanin, phellopterin, and imperatorin, respectively (Singhuber et al. 2011) Imperatorin, together with phellopterin, found in the roots of *A. dahurica*, inhibit [^3^H]diazepam binding to the benzodiazepine site of the rat brain GABA_A_ receptor in vitro with an IC_50_ of 12.3 µmol for imperatorin and 400 nmol for phellopterin. The weaker response at the receptor for imperatorin than for phellopterin is connected with lack of C-5 methoxy group. Since phellopterin was identified as partial agonist of the GABA_A_/benzodiazepine receptor, it is probable that imperatorin, due to the similarity of their structures, has similar properties (Dekermendjian et al. 1996). The other mechanism which increases the GABA concentration in the synaptic cleft, is inhibition of the degradative enzymes, such as GABA transaminase (GABA-T). It was reported that imperatorin, in a concentration ranging from 3.5 to 14 mmol, significantly and irreversibly inhibited GABA-T in a time-dependent and concentration-dependent manner, by irreversibly binding with the active site of GABA-T (Choi et al. 2005).

Another group of very important receptors in the CNS responsible for cognitive funtions are the acetylcholine receptors (AChR) which are stimulated by acetylcholine (ACh). This neurotransmitter is responsible for learning, memory, and cognitive functions (Sarter and Bruno 1997). Acetylcholine is synthesized from acetyl-coenzyme A and choline in a reaction catalyzed by choline acetyltransferase. The degradation of ACh takes place with the participation of the enzyme acetylcholinesterase, and is typical for many neurodegenerative diseases, like Alzheimer’s disease (AD) and Parkinson’s disease (PD) (Purves and Augustine 2004). Imperatorin was examined as an acetylcholinesterase (AChE) inhibitor.

The inhibitory activity was examined in a rapid, bioautographic enzyme assay on thin layer chromatography (TLC) plates. Solutions of pure coumarin derivatives, including ostruthol, ostruthin, imperatorin, and oxypeucedanin hydrate isolated from the roots of *P. ostruthium*, were applied (at 1 µg) on a TLC plate. The minimum inhibitory quantity required to produce white inhibition spots on the TLC plate were calculated as 0.001, 0.06, 0.1 and 0.6 µg, for ostruthol, ostruthin, imperatorin, and oxypeucedanin, respectively. The corresponding values for the standard active control substances galanthamine, physostigmine, and huperzine A were 0.01, 0.005, and 0.002 µg, respectively. Therefore imperatorin shows significant activity against AChE, but is weaker than ostruthol and ostruthin (Urbain et al. 2005). In another in vitro experiment, a variety of plant extracts containing imperatorin as one of the main active compounds was examined. The extracts were tested at concentrations of 500, 1000, and 2000 µg ml^−1^, and the inhibition was measured as a percentage of inhibition (compared with galanthamine). It was concluded that the inhibition was correlated with the occurrence of imperatorin. For example, the stronger inhibition activity (in the range of 10.14–33.85 %) was measured for the methanol extract of the fruits of *Heptaptera anisoptera* (DC.) Tutin (Apiaceae), where imperatorin usually is present in a higher amount, while for the ethyl acetate extracts this activity was weaker (7.09–10.03 %) (Şenol et al. 2010). Imperatorin was also tested as a potential inhibitor of acetyl- and butyrylcholinesterase (BChE). The inhibition activity was evaluated in vitro according to the modified Ellman method. Imperatorin showed selectivity toward BChE rather than AChE, with an IC_50_ value for BChE of 31.4 µmol (vs 9.4 µmol for galanthamine) (Granica et al. 2013).

It has been established that docking in the binding pocket of the enzyme is strictly related to chemical structure of the substrate. Furanocoumarins with a side chain at C-8 and C-5 have inhibitory action against AChE. Compounds without C-5 occupied, but still with a side chain at the C-8 position, like 8-isopentenyloxypsoralen, have a higher affinity for BChE (Granica et al. 2013). These results were partially confirmed by Senol et al. (2011). In the performed experiments, the AChE and BChE inhibitory activities of imperatorin and a crude extract from the fruits of *Angelica archangelica* L. was tested by the spectrophotometric method at concentrations of 12.5, 25, 50, and 100 µg ml^−1^. Molecular docking studies were also performed to establish the interaction between the major coumarins and active gorge of BChE. Imperatorin displayed low inhibition towards AChE (13.75–46.11 %), whereas it had remarkable inhibitory effect against BChE (37.46–83.98 % vs reference galanthamine 80.31 % at a concentration of 100 µg ml^−1^). Pharmacokinetics experiments suggest that imperatorin is a reversible AChE inhibitor, and acts in dose-dependent manner (Senol et al. 2011).

### In vivo studies

Increasing the concentration of ACh in the synaptic cleft has a positive influence on memory and learning processes (Purves and Augustine 2004). In vivo studies on mice established that imperatorin enhances memory as measured by step latency. The experiment was conducted on 10 month-old mice receiving 0.79 mg kg^−1^ of pure imperatorin or 45 % aqueous ethanol extract of *A. archangelica* fruits containing this compound, for 14 days or longer. Groups treated with imperatorin were found to have significantly higher step down latency time (Sigurdsson and Gudbjarnason 2013).

Together with the acetylcholinesterase mechanism, the β-secretase (BACE-1) role should be mentioned as being important in the progression of AD. This enzyme is responsible for β-amyloid protein production in neuronal tissues, which leads to cognitive dysfunction (Vassar et al. 2009). Imperatorin, together with (+)-byakangelicol, were found to be the most effective BACE-1 inhibitors, with IC_50_ values of 91.8 and 104.9 µmol, respectively. The action of the natural furanocoumarins was weaker than that of the synthetic statin-based compound used as a positive control (IC_50_ = 0.2 µmol). However, given the advantages including low molecular weight and facility with crossing the blood–brain barrier following oral or transdermal administration, imperatorin may be a useful lead for further study as a potential treatment for AD (Marumoto et al. 2010). Significant BASE-1 inhibitory activity was noticed also for the metabolites of imperatorin like 6,7-furano-5-prenyloxy hydrocoumaric acid (IC_50_ = 185.6 ± 6.8 µmol) or xanthotoxol (Marumoto and Miyazawa 2010).

The influence of imperatorin on memory impairment, particularly those connected with anxiety and oxidative stress, was tested after acute and chronic administration. The experiment was carried out on Swiss male mice using the elevated plus maze test (EPM), the modified elevated plus maze test (mEPM), and the passive avoidance test (PA). Different doses and different pretreatment times were evaluated. At doses of 10 and 20 mg kg^−1^ and 30 min after injection, imperatorin showed an anxiolytic effect and improved different stages of memory and learning processes—both acquisition and consolidation (Budzyńska et al. 2012). It was also shown that acute administration imperatorin at doses of 10 and 20 mg kg^−1^ reduced the anxiogenic effect of nicotine (0.1 mg kg^−1^, subcutaneous, *s.c.*). Also improvement of cognitive processes was observed following the acute as well as subchronic injections of subthreshold doses of imperatorin (1 mg kg^−1^) and nicotine (0.05 mg kg^−1^) in PA test. Both imperatorin and nicotine influence the cholinergic system—imperatorin as an inhibitor of AChE activity, while nicotine is an agonist of cholinergic receptors. This explains the synergistic effect of the administration of imperatorin and nicotine in low doses (Budzyńska et al. 2014).

Overproduction of free radicals is also associated with neurodegenerative diseases like AD and PD, thus the oxidative stress effects on the whole brain, the hippocampus, and the prefrontal cortex in mice was assessed by determination of the antioxidant enzymes: glutathione peroxidases (GPx), superoxide dismutase (SOD), glutathione reductase (GR) activities, and malondialdehyde concentration (MDA). Imperatorin, administered repeatedly (1 mg kg^−1^, *i.p*., twice daily, for 6 day), inhibited nicotine-induced changes as observed from a lowering of the MDA concentration and increases in the activities of the aforementioned antioxidant enzymes (Budzyńska et al. 2013). In a model of memory impairment induced by scopolamine, the protective properties of imperatorin were examined in cases of oxidative stress and attenuation of cholinergic neurotransmission. Imperatorin was administered acutely (5 and 10 mg kg^−1^) prior to the injection of scopolamine (1 mg kg^−1^) and repeated (7 days, twice daily, 10 mg kg^−1^). In the first case, the furanocoumarin improved memory acquisition and consolidation impaired by scopolamine, and in the second experiment it significantly attenuated the effects of scopolamine on memory acquisition. Increases in the key antioxidant enzyme activities and a decrease in the MDA concentration were also observed (Budzyńska et al. 2013).

Acting like an irreversible GABA-transaminase inhibitor, imperatorin possesses anticonvulsant activity in the mouse maximal electroshock seizure (MES) threshold model. The threshold for electroconvulsions in mice was determined at several times: 15, 30, 60, and 120 min after intraperitoneal *i.p*. administration of imperatorin at increasing doses of 10, 20, 30, 40, 50, and 100 mg kg^−1^. The best anticonvulsant effect was expressed when the compound was injected 30 min before the MES test, with the best result for 100 mg kg^−1^ (increasing the threshold for electroconvulsions by 68 %) (Luszczki et al. 2007a). Imperatorin may synergistically potentiate the anticonvulsant activity of conventional anticonvulsant drugs. Administration at a dose of 40 mg kg^−1^ with carbamazepine, phenytoin and phenobarbital, enhanced their activity in MES model. The effective dose ED_50_ was lowered from 19.6 to 12.2 mg kg^−1^ (by 38 %) for phenobarbital, and from 12.8 to 8.5 mg kg^−1^ (by 34 %) for phenytoin, without a significant potentiation of the total brain concentrations of the synthetic drugs. At 30 and 40 mg kg^−1^*, i.p*. imperatorin significantly potentiated the anticonvulsant activity of carbamazepine against maximal electroshock-induced seizures expressed by lowering the ED_50_ value from 10.8 to 6.8 mg kg^−1^ (by 34 %) and 6.0 mg kg^−1^ (by 42 %), respectively. Moreover, imperatorin at 30 mg kg^−1^ and carbamazepine at 6.8 mg kg^−1^ shows increases the total brain concentration of carbamazepine from 1.260 to 2.328 μg ml^−1^ (by 85 %), which may be caused by modifying the blood-barrier permeability or acting like an inhibitor of multi-drug resistance proteins. The combinations of imperatorin with conventional anti-epileptic drugs neither disturb long-term memory or motor coordination impairment, nor change the neuromuscular grip strength in experimental animals. Therefore, the investigated combinations appear to be both safe and well-tolerated (Luszczki et al. 2007b). In the direct comparison, imperatorin and valproate produced a clear-cut anti-electroshock action in mice, and the experimentally-derived ED_50_ values ranged between 167–290 mg kg^−1^ for imperatorin and 189–255 mg kg^−1^ for valproate, for protecting mice against maximal electroshock induced seizures. An evaluation of the acute neurotoxic effects in the chimney test revealed that the TD_50_ values for imperatorin ranged between 329 and 443 mg kg^−1^ (Luszczki et al. 2009).

### Influence on the cardio-vascular system

Imperatorin was examined as both an antihypertensive and as a cardioprotecting agent. Imperatorin showed a significant reduction in systolic and diastolic blood pressure in mice. The vascular smooth muscle seems to be the target tissue for imperatorin, and probably has the same mechanism of action as typical calcium antagonists like nitrendypine and verapamil, although it is more similar to the phenylalkylamines (Zhang et al. 2011b). The effects of different extracts of the aerial parts of *Angelica dahurica* var. *framosana* on the vascular relaxation of phenylephrine-induced mouse thoracic aorta contraction were investigated. The most active cyclohexane and ethyl acetate extracts were found to have the highest concentrations of imperatorin (4.09 and 1.70 %, respectively) and showed IC_50_ values of 35.3 and 40.5 mg l^−1^, respectively. The effect of imperatorin itself was dose-dependent. Imperatorin, compared to isoimperatorin had a four-fold stronger vasodilatation effect (IC_50_ = 12.2 and 47.6 µmol l^−1^). The vasodilatation effect of imperatorin was significantly attenuated to 24.88 % in the denuded endothelium group compared with the intact endothelium group (Nie et al. 2009). In the phenylephrine-precontracted rabbit corpus cavernosum, imperatorin exhibited a relaxing effect with the IC_50_ 0.85 mmol l^−1^ (Chiou et al. 2001). Imperatorin at a concentration of 10 µmol relaxes the rat mesenteric artery pre-contracted by KCl and endothelin-1, and the human omental artery pre-contracted by noradrenaline. The vasodilation effect was found to be independent of the endothelium (He et al. 2007). The role of calcium-activated potassium channels and ATP-sensitive potassium channel were suggested, inwardly rectifying potassium channel and β-receptor was excluded. The possible mechanism of action is connected with the inhibition of voltage-dependent calcium channel and receptor-mediated Ca^2+^ influx and release (He et al. 2007). In the next study, the role of store-operated calcium entry was excluded (Zhang et al. 2010a). On the other hand, the anti-hypertensive mechanism of imperatorin in rats was connected with its antioxidant effect and the lowering of blood pressure. Treatment with imperatorin (6.25, 12.5, and 25 mg kg^−1^ day^−1^, intragastric *i.g.*) resulted in lowered lipid peroxide levels, as well as an increase in antioxidant enzyme activity. The lowering of lipid peroxide levels was explained by the down-regulation of NADPH oxidase, in effect lowering mRNA expression and the proteins of NADPH oxidase in renal related hypertension (Cao et al. 2013).

Activity against myocardial hypertrophy was also investigated in vitro and in vivo. At 30 μmol imperatorin inhibits cardiac myocyte synthesis induced by angiotensin II and aldosterone, a hormone playing an important role in the pathogenesis of hypertension and myocardial hypertrophy. Imperatorin in the dose range of 3–30 μmol, shows a prevention role and also reduces the existing pathological changes. In consequence, the blood pressure and the heart rate was reduced and the left ventricle wall became thinner, which stopped the process leading to heart failure (Zhang et al. 2010b). Imperatorin also suppressed myocardial protein synthesis increased by isoproprenlol and phenylephrine. Four weeks after low-dose treatment (25 mg kg^−1^ per 24 h, *i.g.*), the myocyte hypertrophy in hypertensive rats was reduced and the hemodynamical parameters were improved. The diameter of the cardiac muscle cells decreased from 51 to 45 µm in the low dose treatment, and from 51 to 31 µm in the high dose treatment. Myocardial fibrosis was reduced by 4 and 12 %, respectively; the myocyte cross-sectional area was also reduced, and the influence on vascular elastic fibers was confirmed. Fibers were arranged in order, and collagen fibers were not significantly hyperplastic in comparison with the control group (Zhang et al. 2010b). Imperatorin (15 mg kg^−1^) also stimulated endothelial nitric oxide synthase (eNOS)—an enzyme responsible for the production of NO, which is important in the process of vasodilation. As a consequence, the blood pressure decreases and cardiac hypertrophy is suppressed, in addition, degenerative changes in myocardial tissue, such as fibrosis are inhibited and ameliorating pulmonary edema prevented (Zhang et al. 2012).

### Antibacterial activity

Furanocoumarins, which are known to possess broad antimicrobial properties, merit further attention to explore their range of activity and potential as lead structures for further development (Stavri and Gibbons 2005). Plants containing imperatorin have a strong justification in bacterial infections in traditional medicine. Imperatorin, together with other coumarins, such as isoimperatorin, phellopterin, byakangelicin, and scopoletin were tested against *Bacillus subtilis*, *Escherichia coli*, *Cladosporium herbarum,* and *Aspergillus candidus.* In most cases, imperatorin was inactive with an MIC of 1000 µg ml^−1^, and weak antimicrobial activity was observed against *B. subtilis* (MIC = 500 µg ml^−1^) (Kwon et al. 2002). Similar results were obtained by Kwon et al. (Kwon et al. 1997). In experiments conducted by Sarker et al. (2007), imperatorin was active against Gram-positive bacteria like *Bacillus cereus* (MIC = 5 × 10^−5^ µg ml^−1^). Imperatorin was also the most active among six coumarins isolated from the acetone and methanol extracts of the flowers of *Magydaris tomentosa* (Desf.) DC (Apiaceae) with MICs between 32 and 128 µg ml^−1^ (Rosselli et al. 2007), as well as among 21 compounds isolated from leaf and stembark extracts of *Clausena anisata* (Will.) Hook. f. ex. Benth. (Rutaceae) with MIC values against *Vibrio cholerae* of 8 µg ml^−1^ and against *Staphylococcus aureus* of 32 µg ml^−1^ (vs chloramphenicol and ampicillin in the range of 8–16 µg ml^−1^) (Tatsimo et al. 2015). In experiments performed by Walasek et al. (2015) imperatorin showed mild activity against Gram-negative bacteria (MIC = 1 µg ml^−1^). High activity for imperatorin against *B. subtilis* (MIC = 0.125 µg ml^−1^), *Candida albicans*, *C. parapsilosis*, *S. aureus* (MIC = 0.25 mg ml^−1^), *B. cereus*, and *M. luteus* (MIC = 0.5 mg ml^−1^) was demonstrated (Walasek et al. 2015). Imperatorin shows strong activity against *Enterobacter cloacae* and *Klebsiella pneumonia*, (MIC 0.028 and 0.030 mg ml^−1^, respectively), stronger than amoxicillin with clavulonic acid (MIC = 0.042 and 0.048 mg ml^−1^, respectively), but weaker than netilmicin (MIC = 0.008 mg ml^−1^) (Widelski et al. 2009). Additionally, it appeared highly active against the oral pathogens *Streptococcus mutans* and *S. viridans* (MICs 0.018 and 0.015 mg ml^−1^, respectively). These studies confirmed the previous theory that the presence of an isoprenyl unit attached to the carbocyclic ring favors the antimicrobial activities of coumarins (Schinkovitz et al. 2003).

### Antiviral activity: influence on replication HIV

Imperatorin was found to be an HIV-1 replication inhibitor. The activity of imperatorin was examined on several cell lines: human cervical carcinoma cell (HeLa), human T leukemia cells (MT-2) and Jurkat T cells at 24, 48, and 72 h and doses of 10, 25, 50, and 100 µmol. In several T cell lines and in HeLa cells, virus-pseudotyped or GP160-enveloped recombinant HIV-1 infection was stopped. Imperatorin apparently operates by two mechanisms—through the Sp1 transcription factor and through cyclin D1 gene transmission. Imperatorin strongly inhibits cyclin D1 expression and arrests the cells at the G1 phase of the cell cycle. HIV-1 replication follows a Sp1-dependent pathway and is directly inhibited by this furanocoumarin (Sancho et al. 2004).

### Anticancer activity of imperatorin

Many papers report the anticancer effects of imperatorin and few mechanisms of this activity were reported, including anoikis, autophagy induction, necrosis, and inhibition of the formation of DNA adducts. The influence of imperatorin on HepG2, SPC-A1, SGC-7901, Bcap-37, MCF-7, HeLa, HT29, HL-60, and K562 cell lines was examined (Luo et al. 2011). Imperatorin significantly reduces HeLa cell and laryngeal carcinoma (Hep-2) cell viability by induction of apoptosis, increasing the activity mediators of apoptosis-caspase-3 and caspase-8 for both cell lines. Simultaneous administration of quercetin and imperatorin (50 and 50 µmol, respectively) for 48 h leads to progressive reduction of HeLa cell viability to 52.86 % and for Hep-2 cells to 39.34 %, while administration of 50 µmol of quercetin and imperatorin alone inhibited cell viability to about 60 and 70.99 %, respectively. Imperatorin alone gave a significant effect only at a concertation of 100 µmol (Bądziul et al. 2014). Jakubowicz-Gil et al. (2012) established that imperatorin is a potent inducer of autophagy, rather than a necrotic or apoptotic one. Comparative studies with cisplatin (2.5 µmol) and imperatorin (50, 100, 150, and 200 µmol) on HeLa cells showed that the furanocoumarin acted as an autophagy inducer alone and in combination with cisplatin. Only very high concentrations of imperatorin (200 µmol) resulted in necrosis. The marker of autophagy was the presence of LC3 cleavage and the inhibition of heat shock proteins expression, especially Hsp27 (Jakubowicz-Gil et al. 2012). Treatment of HepG2 cells with 30 or 60 µmol of imperatorin resulted in increased number of apoptotic cells and lowered pro-caspase expression, when compared to the control (amount of genomic DNA analyzed by electrophoresis) (Luo et al. 2011). The apoptosis mechanism of imperatorin was also shown in HL-60 cells; administration at different concentrations in the range of 0.1–50 µmol reduced viability in a dose-dependent manner (100 % for control) to almost 100 % for 0.1 µmol of imperatorin and to 28 % for the highest concentration of the tested compound. Fragmentation and nuclear morphological changes, characteristic for apoptosis, and an increase of caspase-3 and caspase-9 activity, not caspase-8 were observed, which suggested a mitochondrial, not a receptor-mediated, pathway of apoptosis (Pae et al. 2002). A combination of imperatorin and quercetin (50 and 100 µmol, respectively) also decreased the level of heat shock proteins Hsp27 and Hsp72 (Bądziul et al. 2014). After treatment with 30, 45, 60, and 90 µmol of imperatorin, the level of the anti-apoptotic protein—Bcl-2, decreased and the levels of the pro-apoptotic proteins Bax, Bad, and tBid were increased (Western- blot analysis, β-actin as control). In a dose-dependent manner (dosage as above), significant lowering in the levels of pro-caspase-3, pro-caspase-8, and pro-caspase-9 occurred (Western blot, β-actin as control). With an increasing concentration of imperatorin, the expression of death receptors, known as the TNF superfamily receptor 6 (Fas protein), Fas-associated protein with death domain (FADD), p21 and p53 increases (Luo et al. 2011). Imperatorin at sub-toxic concentrations (0.1, 0.5, 1, 5, and 10 µg) sensitized lung cancer cell for anoikis and inhibited the growth of cancer cells in the detached condition. Additional significant enhancement of the p53 protein levels and the proteins from the Bcl-1 family suggests a possible anti-metastasis role for imperatorin, which may be beneficial for cancer therapy (Choochuay et al. 2013). It was reported that programmed cell death takes place in the G1/S transcription phases of the cell cycle, which means it is directed towards growing cells (Appendino et al. 2004).

Imperatorin significantly blocked the activity of the polycyclic aromatic hydrocarbon reactive metabolites 7,12-dimethylbenz[*a*]anthracene (DMBA) and benzo[*a*]pyrene B[a]P. These metabolites form DNA adducts which are responsible for tumor growth (Cai et al. 1997). Imperatorin inhibited DMBA and B[a]P bioactivation in several cell lines: in MCF-7 human breast cancer cell line by 58 and 63 %, respectively, at a concentration 10 µmol (Kleiner et al. 2003), on the SKOV-3 (ovary) and XF498 (central nerve system) cell lines imperatorin showed very good results with ED_50_ 13.1 ± 0.3 and 12.3 ± 0.5 µg ml^−1^, respectively (Kim et al. 2007). In the case of mouse mammary (Prince et al. 2009) and skin tumors in cultured mouse keratinocytes, imperatorin acted most effectively at 150 and 70 mg kg^−1^, respectively (Kleiner et al. 2002). The interesting fact is the low toxicity of imperatorin, even when applied at the high doses (5, 10, 20, 80 µmol) (Kleiner et al. 2003).

Imperatorin in low and high dose treatment (50 and 100 mg kg^−1^, *per osp.o*., 14 days) suppresses tumor growth in HepG2-bearing nude mice, without significant side effects, such as weight loss, cardiotoxicity, or hepatoxicity. The tumor size was significantly decreased by 31.93 and 63.18 % for the low dosage and high dosage treatments, respectively (Luo et al. 2011). Imperatorin also induces the activities of carcinogen-detoxifying enzymes, such as glutathione *S*-transferase (GST) and oxidoreductase (NQO1). The activity increased from 9.5 nmol min^−1^ mg^−1^ protein (control) to 13 nmol min^−1^ mg^−1^ protein for NQO1 and from 10.9 to 34.54 nmol min^−1^ mg^−1^ protein for GST with imperatorin doses in the range of 6.25–200 µmol for 24 h) (Prince et al. 2009).

### Other pharmacological properties

Imperatorin was examined as a bone loss inhibitor. It improved osteo-induction by increasing BMP-2 gene expression more than threefold at a dose of 10 µmol compared with control. Collagen content in the tissue and mineralization were also increased with a higher concentration of imperatorin (Chen 2011). The cell osteoblast activity compared to control was almost doubled in treatment with 10 µmol of imperatorin. Also, the collagen content raised more than two-fold when the control value was 100 % at 10 µmol of imperatorin. The percent of mineralized nodule was raised by almost 225 % when the control value was 100 % (Chen 2011).

Another interesting property of imperatorin is the hepatoprotective action. Imperatorin was found to inhibit the elevation of plasma alanine aminotransferase (ALT) in mice with concanavalin A-induced hepatitis and mice anti-Fas antibody-induced hepatitis. At a dose of 100 mg kg^−1^, 83 % of this induced elevation of ALT activity was inhibited (Okamoto et al. 2001).

Potent myorelaxant activity of imperatorin was examined in rat isolated jejunum strips. In the dose range 0.001–100 µmol, gradual myorelaxation was observed. At 25 and 50 µmol imperatorin caused relaxation comparable to the strength of the reaction induced by isoproterenol at 0.1 µmol. The mechanism of this action is associated with various Ca^2+^ influx pathways (Mendel et al. 2015).

Imperatorin was also examined for its effects on macrophage functions of relevance to the inflammatory process. Ionophore-stimulated, mouse peritoneal macrophages served as a source of cyclooxygenase-1 (COX-1) and 5-lipooxygenase (5-LOX). The suggested mechanism was connected with the release of COX-1 and 5-LOX enzymes and products of its catalytic effects—prostaglandin 2 (PGE_2_) and leukotriene 4 (LTC_4_) and the activity of COX-2 (cyclooxygenase 2) and nitric oxide synthase (NOS) induced by *E. coli* lipopolisaccharydes. With an IC_50_ < 15 µmol, imperatorin showed a significant effect on 5-LOX release. Imperatorin exhibited strong to medium inhibition of PGE_2_ release in the dose range 25–100 µmol. and strongly reduced LTC_4_ generation (1–100 µmol). In a dose-dependent manner imperatorin (in the range of 25–100 µmol) inhibited the LPS-induced PGE_2_ release measured in ng ml^−1^ went down from 1.13 to 0.13 respectively, an effect related to COX-2 activity. Imperatorin (IC_50_ = 9.2 µmol) was also effective as an inhibitor of NO synthesis. The relative selectivity ratio COX-1/COX-2 was 0.90 indicating an almost equipotent effect of imperatorin at 100 µmol. It is very probable that the COX-2 inhibitory effects are connected with the substitution at positions 5 and 10 in the furanocoumarin structure (Abad et al. 2001).

## Pharmacokinetics of imperatorin

The kinetics of a drug describes its behavior in an organism in five steps: liberation, absorption, distribution, metabolisation, and excretion. The liberation step is important with respect to the pharmaceutical presentation of the drug, and has technological meaning, which is not a key aspect of this paper. The absorption process after the oral administration of imperatorin in rats was described as slow and the bioavailability as poor (Zhao et al. 2013), however the absorption of an extract (95 % ethanol) of Radix *Angelicae pubescentis* was rapid, which was explained as due to the synergistic action of other coumarins occurring in the plant material (Chang et al. 2013). After *i.v.* administration of imperatorin, the extravascular distribution was low, and the compound was rapidly eliminated. High values for the plasma protein binding, which reached more than 90 %, could account for the slow distribution into the intra- and extracellular spaces. Correlation coefficient values suggested linear pharmacokinetics, however there were significant, dose-dependent differences in the pharmacokinetic parameters (Zhao et al. 2013). Two metabolites of imperatorin, as a products of phase I reactions taking place in liver microsomes, were isolated resulting from demethylation and oxygenation. The demethylation and oxygenation reactions lead to the formation of xanthotoxol and heraclenin. The heraclenin was detected and identified in rat plasma, whereas the xanthotoxol and heraclenin were identified in rat urine. There were no metabolites detected in the bile and feaces (Zhao et al. 2013).

Further studies concerning the metabolites of imperatorin, found in urine of rats, provided an exceptionally complete pattern of the processes and products of the phase I and II reactions. Those reactions of phase I included: mono-, di-, and tri-oxidation, internal hydrolysis, mono-oxidation and di-oxidation combined with internal hydrolysis, demethylation to a carboxylic acid, loss of the C_5_H_8_ group combined with oxidation and methylation. In the phase II reactions: sulfate conjugation, oxidation with sulfate conjugation, di-oxidation, and glucuronide conjugation. These processes afforded fifty-one identified metabolites of imperatorin (Qiao et al. 2013).

It was established that imperatorin induces phase I and phase II enzymes. Increased activity of the P450 cytochromes and glutathione *S*-transferases (GSTs), two important enzyme families involved in metabolism, were observed. The maximum induction of GST activities (using CDNB—1-chloro-2.4 dinitrobenzene, DCNB—1,2-dichloro-4-nitrobenzene, and EA—ethacrynic acid as substrates) by imperatorin were 260, 300, and 170 % of the corn oil control, respectively (Kleiner et al. 2008).

Further studies showed that imperatorin can inhibit P4501A1-mediated ethoxyresorutin *O*-dealkylase (EROD) activity in hepatic microsomes without a major effect on heme content. Three potential mechanisms of these inactivation are possible, including—alkylation of the P450-prosthetic heme group, degradation of the prosthetic heme group, and covalent binding to the apoprotein. However, the specific mechanism of the inactivation of P450-mediated EROD activity has not been determined for furanocoumarins (Cai et al. 1996). Imperatorin was found to be a competitive inhibitor of EROD, and was unable to inactivate microsomal PROD; evidence of some selectivity (Cai et al. 1993). These results confirm earlier studies conducted on mice at Seoul National University, where it was observed that there was a significant reduction in the activity of microsomal mixed function oxidases, such as hepatic aminopyrine *N*-demethylase, hexobarbital hydroxylase, and aniline hydroxylase. In the first case, imperatorin was shown to be a competitive inhibitor, and in the second and the third instances, a non-competitive inhibitor (Cai et al. 1993).

A very important issue in determining the pharmacokinetic properties of a drug is establishing its ability to cross the blood–brain barrier (BBB). As a relatively non-polar molecule, imperatorin passes easily through the BBB and is a highly permeable compound. Two mechanisms for this process were considered: transcellular passive diffusion and active transportation, which involves influx and efflux transporters. The P-gp protein (permeability glycoprotein) is a product of the multidrug resistance (*mdr*) gene and it is considered that PGP functions as an efflux system at the BBB. The PGP protein acts as an adenosine triphosphate–dependent drug efflux system in cells. Thus disruption of the PGP gene in mice leads to increased uptake of PGP substances into the brain. That suggest this protein has a very important function in BBB permeability, and that dysfunction of PGP makes drug-drug interactions more probable (Matsuoka et al. 1999). Imperatorin is not a substrate for the P-gp protein and does not inhibit its activity (Lili et al. 2013). Imperatorin has different distribution in various rat brain tissues. The highest concentrations were detected in the striatum, hippocampus, brain stem, cortex, cerebellum, and diencephalon, respectively. The half-life in the hippocampus was significantly longer than in other structures (Zhang et al. 2011a).

## Analytical aspects of the isolation and identification of imperatorin

### Extraction of imperatorin from plant material

Obtaining the pure target substance from a complex plant matrix for biological evaluation remains a challenging, and frequently a very problematic step, and is a significant barrier in natural product development. The selection of extracting solvent and the method, time, energy requirements of the extraction process depends on a variety of factors, including the polarity and the stability of the compound of interest, the relative concentration in the matrix, and the properties of co-metabolites. The first step is the extraction of crude sample with solvent—known as liquid–solid extraction (LSE).

The common solvents used for the isolation of coumarins are ethanol, methanol, dichloromethane, ethyl acetate, diethyl ether, petroleum ether, and their combinations (Skalicka-Woźniak and Głowniak 2014). In order to select the most efficient process to obtain pure imperatorin, a variety of extraction methods were evaluated, including Soxhlet extraction (SX), ultrasound assisted extraction (UAE) at room temperature and at 60 °C, microwave assisted extraction (MAE) in open and closed systems, or accelerated solvent extraction (ASE). For the isolation of imperatorin from the fruits of *Peucedanum verticalle* (L.) Koch ex DC. (Apiaceae), the best results were achieved with UAE at 60 °C with petroleum ether as solvent (6.68 mg g^−1^ compared to 2.05 mg g^−1^ at 20 °C) (Waksmundzka-Hajnos et al. 2012). When closed and open microwave assisted extraction was used, different effects were observed. Extraction in 80 % methanol with a higher pressure resulted in a decrease of imperatorin obtained from 3.59 to 2.02 mg g^−1^ (Waksmundzka-Hajnos et al. 2012).

The most effective process for furanocoumarin extraction was determined to be ASE, especially for the more hydrophobic metabolites, such as imperatorin and phellopterin. This extraction methodology was employed when the fruits of *Archangelica officinalis* L. (Apiaceae) were examined. Extraction with petroleum ether, followed by methanol (100 °C, 60 bar) yielded 19.08 mg g^−1^ of imperatorin, and a similar efficiency (18.86 mg g^−1^) was observed with MAE when an 80 % solution of methanol was used (Waksmundzka-Hajnos et al. 2004a). The simplest methods, SX and UAE (extraction with petroleum ether, followed by methanol), gave significantly lower yields of 13.12 mg g^−1^ for SX, and 12.75 and 14.00 mg g^−1^ for USE at 25 and 60 °C, respectively (Waksmundzka-Hajnos et al. 2004a). On the other hand, for the isolation of imperatorin from the fruits of *Pastinaca sativa* L. (Apiaceae) ASE yielded 15.12 mg g^−1^, while UAE at 60 °C 14.44 mg g^−1^ (Waksmundzka-Hajnos et al. 2004b). Thus each plant matrix requires exploration to discern the most effective extraction protocol.

ASE was also applied for the extraction of the fruits of *Heracleum leskowii* L. (Apiaceae). Different conditions of temperature (80, 90, 100, and 110 °C) and solvent (methanol, ether, and dichloromethane) were evaluated. The results indicated that dichloromethane was slightly better for imperatorin extraction than methanol and better than ether. Increasing the temperature effected an increase in the extraction yield of imperatorin (17.87, 18.06, 18.22, and 19.07 mg 100 g^−1^ at temperatures 80, 90, 100, and 110 °C, respectively). The SX method was very unsatisfactory due to the thermal degradation of furanocoumarins (De Castro and Da Silva 1997; Skalicka-Woźniak and Głowniak 2012).

An alternative method used to achieve the almost complete separation of imperatorin from the plant matrix is supercritical fluid extraction (SFE). Imperatorin, together with meranzin and meranzin hydrate, were extracted under the same conditions (50 °C and 27.6 MPa). The extraction efficiencies were 84, 76, and 18 %, respectively. The solubility of coumarins in CO_2_ in the absence of a modifier decreases as the polarity of the functional groups on the coumarins increases. Therefore substitution of a functional group tended to reduce its solubility in the order methyl>methoxyl>hydroxyl (Choi et al. 1998). In a simplified way, the supercritical CO_2_ process is a non-polar technique, and consequently it is no surprise that the addition of polar solvent, such as ethanol, improves the efficiency of extraction when it is considered that substituted coumarin derivatives are less soluble in supercritical CO_2_ than simple coumarins (Choi et al. 1998). For the efficiency of supercritical CO_2_ extraction, different parameters, including pressure, temperature, flow rate of CO_2_, and the amount of modifier, have an undoubted influence. The optimal conditions for the extraction of the coumarins from the dried roots of *A. dahurica* by SFE were 30 MPa of pressure, 50 °C, 2.0 l min^−1^ of flow rate for CO_2_, and 4 l min^−1^ for the flow rate of 75 % ethanol as a modifier. The relative content of imperatorin extracted with this method (8.96 %) was the highest compared with the other coumarins. In comparing the three extraction methods, solvent extraction SE, UAE, and SFE, the best effects, a higher concentration of coumarins with fewer impurities was obtained when the SFE method was used. The quantitative term used is the relative impurity content (RIC), which is defined as the amplitude of the total peak area, except for imperatorin, cnidilin, and isoimperatorin, to the sum of the peak area of the enumerated coumarins. The lower is the RIC, the purer is the extract. From the described methods, the RIC values for SE, UAE, and SFE were as follows: 1.51 ± 0.1, 0.89 ± 0.03, and 0.45 ± 0.02, respectively (Wang et al. 2011). Imperatorin was also obtained with good results from the peel of *Citrus maxima* (Burm. f.) Merr (Rutaceae). The highest extraction efficiency was obtained at 50 °C and 27.6 MPa in a yield of 1.28 mg g^−1^ of imperatorin; ethanol was selected as the best modifier (Teng et al. 2005).

### Isolation and purification

Plant extracts are typically mixtures of a large number of active constituents with similar structures and different polarities. Obtained extracts are rich in chlorophylls (whole plant and leaves), oils and waxes (fruits and semen), carbohydrates (roots), which have different polarities, thus an important step is selection of the proper isolation technique. However, even the optimal method of extraction will leave impurities in a sample. Column chromatography, often followed by preparative thin layer chromatography and counter-current chromatography are the typical techniques used for the isolation of imperatorin from a crude plant extract.

#### Column chromatography

Column chromatography is a very popular technique for the separation and purification of target compounds, and is still very often the first step in sample partition. A number of different absorbents and solvent systems may be tested to obtain the separation of the target compound. The most widely-used absorbents are: aluminum oxide, silica gel, and Florisil. On aluminum oxide (activity degree III), imperatorin can be separated using petroleum ether and chloroform (2:1), chloroform, and chloroform-ethanol mixtures (9:1, 4:1, and 2:1) as eluents, depending on the complexity of the starting matrix. When silica gel is used, mixtures of hexane-chloroform and chloroform-ethanol with increased proportions of a more hydrophilic component are used (Lozhkin and Sakanyan 2006; Skalicka-Woźniak and Głowniak 2014). For example, in the gradient elution (petroleum ether:acetone) of a 95 % ethanolic extract from the roots of *A. dahurica* on silica gel, imperatorin was identified in fractions eluted with petroleum ether:acetone (100:5). The fraction was further separated on an open ODS column with mixtures of methanol and water. Imperatorin was obtained in the fractions eluted by a 48:52 (*v*/*v*) methanol:water system. From a 1 g sample of plant material 0.35 mg of pure compound was obtained (Wang et al. 2007). The methylene chloride soluble extract (70 g) from the same plant was also chromatographed on silica gel. Stepwise gradient elution with mixtures of hexane and ethyl acetate (5:1–0:1) resulted in six fractions. After rechromatography using the same conditions of fraction 4, imperatorin was obtained (Piao et al. 2004). In another example, the ethyl acetate layer from an 80 % aqueous methanol extract of the roots of *A. dahurica* was chromatographed on a silica gel column with the gradient elution with *n*-hexane-ethyl acetate with increasing proportions of ethyl acetate afforded eleven subfractions. Imperatorin was obtained by silica gel column chromatography of subfraction 8 using *n*-hexane: chloroform:ethanol (25:20:1) as the eluting solvent. The separation of a sample of 36 g of the crude extract yielded 628 mg of imperatorin (Baek et al. 2000).

Also very useful for the separation of non-polar extracts is Florisil. One example of the successful application of Florisil is the isolation of imperatorin from a petroleum extract of the fruits of *Heracleum mantegazzianum* Sommier & Levier (Apiaceae). The first column was eluted with a gradient of dichloromethane and ethyl acetate, increasing the latter solvent from 0 to 20 %. The first four fractions richest in coumarins were consolidated and re-chromatographed using the same conditions, and the first two fractions were subject to a column filled with silica gel 60 with a gradient of dichloromethane:ethyl acetate, increasing the latter solvent from 0 to 10 %. This separation resulted in a mixture of furanocoumarins, but not pure imperatorin (Glowniak et al. 2000).

#### Preparative thin layer chromatography (prep-TLC)

Prep-TLC was used by Zgórka and Głowniak (1993) for the isolation of imperatorin from the fruits of *Libanotis dolichostyla* Schischk. (Apiaceae). The plant material was first extracted with petroleum ether, and then chromatographed on successive columns of silica gel and Florisil. The mixtures of coumarins obtained were subjected to prep-TLC in order for final purification (Zgórka and Głowniak 1993; Cisowski et al. 1991) applied prep-TLC of coumarins using gradient elution on the chloroform extract (0.4 g) of the roots of *P. ostruthium* dissolved in a mixture of acetone and methanol (5 ml, 6:4 *v*/*v*) on a TLC plate developed as follows: chloroform-ethyl acetate (95:5 *v*/*v*); 90:10:85:15; chloroform-ethyl acetate-methanol 85:15:2; 85:15:5, and 85:15:7. Using this protocol 15 mg of pure imperatorin was isolated (Cisowski et al. 1991).

#### Counter-current chromatography

Counter-current chromatography (CCC) is a liquid–liquid chromatographic technique which effects the isolation of substances with high purity from a crude extract, based on their partition coefficient between two liquid phases. In this technique, the biphasic system of solvents is subjected to the effects of gravitation and centrifugal force to retain one phase in a coil, when the second immiscible phase is passed through. As a result of the specific planetary motion, the sample travels through the coil where mixing and settling of the biphasic system occurs. Substances with a high affinity for the stationary phase are retained within the coil. Those substances which are more soluble in the mobile phase pass through and are eluted. The technique has a number of significant advantages over traditional solid-phase chromatography systems: the solvent consumption is generally far lower than that of solid phase chromatography and there is no loss of complex matrix material arising from irreversible adsorption onto the solid matrix. The most important step in this technique is choosing an appropriate solvent system (Skalicka-Wozniak and Garrard 2015). Different analytical conditions, as well as the two-phase solvent systems which have been used for the purification of imperatorin, together with their efficiency, are described in Table 3.

**Table 3:** Application of CCC in isolation of imperatorin

Plant material and the amount of extract subjected to purification	Two-phase solvent system	Amount and purity of isolated imperatorin	References
*A. dahurica*—root 95 % Ethanol and ethyl acetate extract 300 mg	Multidimentional CCC: *n*-Hexane-ethyl acetate-methanol-water 1:1:1:1 and 5:5:4.5:5.5	19.9 mg 98 %	Wei and Ito (2006)
*A. dahurica*—herb Ether extract 100 mg	Gradient extraction: *n*-Hexane-methanol-water 5:5:5–5:7:3	29 mg 99.1 %	Liu et al. (2004a)
*A. dahurica*—root 95 % Ethanol and ethyl acetate extract 300 mg	*n*-Hexane-ethyl acetate-methanol-water 5:5:5:5 Then *n*-hexane-ethyl acetate-methanol-water 5:5:4:6 for further purification	18.5 mg 98 %	Wei et al. (2009)
*A. officinalis*—fruits Methanol extract 800 mg	Heptane-ethyl acetate-methanol-water 1:1:1:1	95 mg 99 %	Budzynska et al. (2014)
*C. monnieri*—fruits Ethanol extract 500 mg	Stepwise elution: *n*-Hexane-ethyl acetate- ethanol–water 5:5:4:6–5:5:6:4	118.3 mg 98.2 %	Li and Chen (2004)
*C. monnieri*—whole plant Ethanol and petroleum ether extract 150 mg	Stepwise elution: Petroleum ether-ethyl acetate-methanol 5:5:5:5, 5:5:6:4 and 5:5:6.5:3.5	60.5 mg 100 %	Liu et al. (2004b)
*P. osthrutium*—root Dichloromethane extract 3 g	*n*-Hexane-ethyl acetate-methanol-water 10:5:5:1 Then *n*-hexane-*t*-butyl methyl ether-acetonitrile 5:2:4 for further purification	56 mg Compound purity checked by TLC and HPLC. Values not provided	Urbain et al. (2005)

## Analytical methods

### Thin layer chromatography

Thin layer chromatography (TLC) is a very popular and effective tool used for analytical and quantitative purposes. The separation depends on balance between the affinity of the compound to stationary phase and mobile phase. As a result of the simplicity of the technique, TLC is often a first step in preliminary compound identification. For the analysis of furanocoumarin, several adsorbents (silica gel, C_18_ layers, alumina, polyamide, Florisil) (Cieśla and Waksmundzka-Hajnos 2009) and solvent systems (benzene-acetone (90:10, *v*/*v*); toluene-acetone (95:5, *v*/*v*); benzene-ethyl acetate (9:1, *v*/*v*); benzene-diethyl ether-methanol-chloroform (20:1:1:1, *v*/*v*); chloroform, and ethyl acetate-hexane (25:75, *v*/*v*) were evaluated (Bogucka-Kocka 1999). There are many examples of the efficient combination of adsorbents and solvent systems for the identification of imperatorin, and some of them are presented in Table 4.

**Table 4:** Examples of the analysis of imperatorin in plant extracts by TLC

Plant extract	Adsorbent	Solvent system	Detection	References
*One dimensional TLC*				
*A. officinalis* petroleum ether extract	HPTLC LiChrospher Si 60 F_254S_	Dichloromethane:heptan (1:11 *v*/*v*) + 40 % ethyl acetate	313 and 365 nm	Hawryl et al. (2000)
*Angelica sinensis, A. dahurica* and *A. pubescens*—roots (comparative study) *n*-hexane or dichloromethane extracts:	Silica gel 60 F_254_	Toluene:ethylacetate:glacial acetic acid (9:1:0.1)	254 and 366 nm and after spraying with anisaldehyde/sulfuric acid reagent	Zschocke et al. (1998)
*L. dolichostyla*—fruits petroleum ether extract	Silica gel	*n*-Hexane:dichloromethane (1:1 *v*/*v*) + 20 % ethyl acetate	365 nm	Zgórka (2001)
*L. dolichostyla*—fruits petroleum ether extract	Florisil	*n*-Hexane:dichloromethane (1:1 *v*/*v*) + 20 % butyl acetate	365 nm	Zgórka (2001)
*Two-dimensional TLC*				
*Heracleum sibiricum*—fruits petroleum ether extract	Silica gel (Merck)	1st direction benzene: chloroform: acetonitrile (1:1 *v*/*v* + 5 %)	0.5 % I dissolved in KI Dragendorf’s reagent 25 % SbCl_5_ in CCl_4_ UV	Bogucka-Kocka (1999)
		2nd direction benzene:ethyl acetate (1:1 *v*/*v* + 5 %) when separation was unsatisfactory again in 1st direction benzene:chloroform:ethyl acetate (1:1 *v*/*v* + 5 %)		
Coumarin mixtures isolated from plants in the genus *Angelica*	TLC and HPTLC silica gel 60 F_254_ plates	1st direction chloroform (100 %) for 55 min 2nd direction *n*-hexane: ethyl acetate (7:3 *v*/*v*) for 80 min	254, 366 nm	Härmälä et al. (1990)

Some interesting experiments on the separation of coumarins were performed by Waksmundzka-Hajnos et al. (2006). Two-dimensional TLC on polar-bonded stationary phases (CN-silica and Diol-silica) in either non-aqueous (in adsorption mode) or aqueous systems (in partition mode) was used for the separation of closely related coumarins in the fruits and roots of *A. officinalis* and *Heracelum* spp. Through the use of 2D-TLC on CN-silica using the systems: 30 % acetonitrile:water and 35 % ethyl acetate:*n*-heptane, imperatorin could be completely separated from other coumarins. In addition, using the multiphase system C_18_-silica (connected strips) with 55 % methanol–water (on C_18_) and 35 % ethyl acetate:*n*-heptane (on silica) enabled the complete separation.

### HPLC analysis

Currently, HPLC analysis is a standard analytical procedure in phytochemical investigations. This chromatographic technique is widely used for the qualitative and quantitative analyses of furanocoumarins. Before the sample is subjected to HPLC analysis some preparation is typically necessary. The most effective method is solid phase extraction (SPE), the essence of which is the adsorption of the target compounds on prepared SPE microcolumns and then washing them with an appropriate solvent. In most cases, the extract is dissolved in aqueous methanol and passed through conditioned columns and then eluted with 60–80 % methanol in order to afford imperatorin (Zgórka and Głowniak 1993; Sidwa-Gorycka et al. 2003; Bartnik and Głowniak 2007). Oniszczuk et al. (2013) used the SPE method for the purification of different extracts from the fruits of *H. leskowii* (petroleum ether, 80 % aqueous solution of methanol, dichloromethane, and petroleum ether:methanol 50:50, *v*/*v*). The authors compared a novel application of matrix solid-phase dispersion (MSPD) methodology and classical liquid–solid extraction (LSE) connected with solid-phase extraction (SPE) for the determination of furanocoumarins. The samples were dissolved in 80 % aqueous methanol (10 ml) and 2.5 ml was passed through a Baker-bond C_18_ SPE column. The retained furanocoumarins were eluted with 80 % aqueous methanol (10 ml). Either for LSE or MSPE 80 % aqueous methanol gave the best extraction yield of imperatorin (381.42 and 361.06 mg 100 g^−1^, respectively). The recoveries for both methods were in the range of 92.43–96.27 % and 94.04–102.31 % for MSPD and LSE, respectively (Oniszczuk et al. 2013).

Usually a reversed phase system (RP) is the method of choice for the HPLC analysis of imperatorin. Mixtures of methanol or acetonitrile with water are the most commonly used eluents. Rarely, there is a small addition of acid. Because acetonitrile and methanol may have different selectivity in the analysis of complex matrices, the mixture of acetonitrile, methanol, and water is sometimes ultimately determined as the mobile phase (Skalicka-Woźniak and Głowniak 2014). The most useful combinations of solvent systems for the HPLC analysis of imperatorin are indicated in Table 5.

**Table 5:** The most useful combinations of solvent systems for the HPLC analysis of imperatorin

Plant extract	Adsorbent	Solvent system	Flow rate (ml min^−1^)	Retention time (min)	References
*A. dahurica* roots ethanol extract	Kromasil C_18_ 250 × 4.6 mm; 5 µm column with 5 µm C-18 guard	Methanol:water 66:34 (*v*/*v*) (isocratic mode)	0.8	18	Wang et al. (2007)
*A. dahurica* roots ethanol extract	Polaris ODS 250 × 4.6 mm ID	Methanol:water 60:40 (*v*/*v*) (isocratic mode)	1	10	Wei et al. (2009)
*A. dahurica*roots different extracts	YMC ODS-C_18_ column 250 × 4.6 mm; 5 µm	Linear gradient elution: 20–24 % A (0–10 min); 24–40 % A (10–40 min); 40–52 % A (40–60 min); 52–72 % A (60–90 min)—A (acetonitrile-methanol = 5:3) and B—water	1	43.07	Xie et al. (2010)
*C. monnieri* fruits micelle-mediated extract	Zorbax SB-C18 column 4.6 mm i.d. × 150 mm, 5 µm	Gradient: 10–30 % over 0–15 min, 30–90 % over 15–30 min, 30 % at 30 min of 0.1 % phosphoric acid and acetonitrile	1	12	Zhou et al. (2008)
*C. maxima* peel of fruits SFE-CO_2_	Merck Chromolith™ RP-18e column 100 × 4.6 mm	Gradient elution: 20–80 (A:B) for 5 min, followed by a linear gradient to 28–72 in 0.5 min, maintained at 28–72 for 6.5 min, followed by a linear gradient to 30–70 in 0.5 min, maintained at 30–70 for 7.5 min, followed by a linear gradient to 32–68 in 0.1 min, and maintained at 32–68 for 40 min. A—acetonitrile and B—water containing 1 % (*v*/*v*) acetic acid	2	51.16	Teng et al. (2005)
*H. leskowii* fruits petroleum ether, dichloromethane and methanol extracts	Hypersil BDS C_18_ 250 × 4.6 mm; 5 μm	Gradient: 50–60 % A (0–5 min); 60–80 % A (5–25 min); isocratic 80 % A (25–30 min); 80–100 % A (30–40 min). A—methanol, B—water	1	16.74	Skalicka-Woźniak and Głowniak (2012)
*L. dolichostyla* fruits methanol extract	Hypersil ODS column (200 mm × 4.6 mm; 5 μm)	Acetonitrile: water 60:40 *v*/*v*) (isocratic mode)	1	Not provided	Zgórka and Głowniak (1993)
*P. sativa* and *A. officinalis* fruits petroleum ether and methanol extracts	SUPELCOSIL™ LC_18_ 150 × 4.6 mm; 5 μm	Gradient elution: 45 % methanol (0–10 min); 45–55 % methanol (10–20 min); 55–70 % methanol (20–30 min); 70 % methanol in water (30–40 min)	1	32	Waksmundzka-Hajnos et al. (2004a, b)
*P. osthruthium* roots methanol extract	Zorbax Rx C_18_ 250 × 4.6 mm; 5 μm	Gradient: 85 % water (3 min), 80 % water (2 min), 70 % water (10 min), 55 % water (3 min), 50 % water (7 min), 30 % water(10 min), 15 % water (3 min), 5 % water (5 min). Water: acetonitrile solvent with 0.01 % acetic acid or 1 mM NH_4_OAc	1	35	Ganzera et al. (1997)
*P. ostruthium* roots dichloromethane extract	RP-_18_ 150 × 4 mm; 3.9 μm	Gradient of methanol and water: 25–75 to 100–0 containing 0.05 % trifluoroacetic acid during 25 min, followed by 5 min with 100 % methanol	1		Urbain et al. (2005)
*P. palustrae* and *A. officinalis* root methanol extract	RP-_18_ prodigy 100 × 4.6 mm; 3.0 μm	Methanol: 1 % formic acid 60:40 (*v*/*v*)	1	23.1	Eeva et al. (2004)

### Gas chromatography

Coumarins are also detectable by gas chromatography. Investigations of the retention times of substituted furanocoumarins is revealed the following general principles: (1) the retention time is lower for methoxycoumarins than hydroxycoumarins because of the reduced adsorption due to hydrogen bonding; (2) furocoumarins with *O*-alkyl substituents at C-5 are eluted after the 8-hydroxy isomers; (3) the logarithm of the relative retention time is a linear function of the molecular weight. This GC data can be used for assisting in determining the structure and estimating the retention times of analogous coumarins (Lozhkin and Sakanyan 2006). For imperatorin, the GC method was validated for a SFE extract of the fruits of *C. monnieri*. GC–MS with a DB-5MS capillary column (30 m × 0.32 mm i.d. 0.25 µm film thickness) was used. The inlet temperature was maintained at 280 °C. The oven temperature was initially held at 60 °C for 2 min, and then programmed to 280 °C at 10 °C min^−1^, and held constant for 6 min. Helium was used as a carrier gas at a constant flow rate of 2.0 ml min^−1^. With these parameters, the retention time for imperatorin was equal 20.22 (Chen et al. 2009). GC–MS was used as a method of choice for the simultaneous determination of imperatorin, together with other known coumarins, in rat plasma and tissues. The retention time for imperatorin was 12 min using following parameters: a DB-5MS capillary column, as above, the inlet temperature was maintained at 280 °C. The column oven was held at 140 °C for 2 min, then programmed from 140 to 280 °C at 10 °C min^−1^, and finally held for 4 min. Helium at a constant flow rate of 0.2 ml min^−1^ was used as the carrier gas (Wang et al. 2009).

## Methods of identification: UV-DAD, MS, NMR spectra

**Fig. 3:**
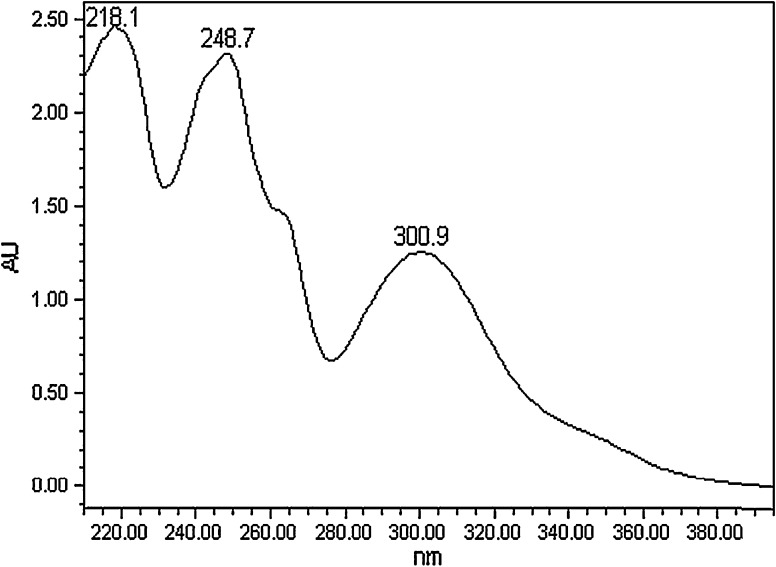
UV–DAD spectrum of imperatorin (Kim et al. 2011)

Imperatorin possesses distinctive chromophore groups which are conveniently observed under UV light, and their UV absorption spectra are also useful in their structure determination. Specific absorption bands at 274 and 311 nm, attributed to benzene and pyrone rings, respectively, are observed. Methyl substitutions at C-5, C-7, and C-8 lead to a bathochromic shift of the 274 nm peak, but not of the 311 nm peak. Linear furanocoumarins, including imperatorin, show four areas of absorption, at 205–225, 240–255, 260–270, and 298–316 nm (Murray and Mendez 1982; Skalicka-Woźniak and Głowniak 2014). UV λ_max_ values obtained from the HPLC–DAD analysis of imperatorin are 217.9, 248.8, 263.5, and 302.9 nm (Liu et al. 2011), or 218.1, 248.7, and 300.9 nm (Kim et al. 2011). The characteristic UV–DAD spectra for imperatorin are presented in Fig. 3.

**Fig. 4:**
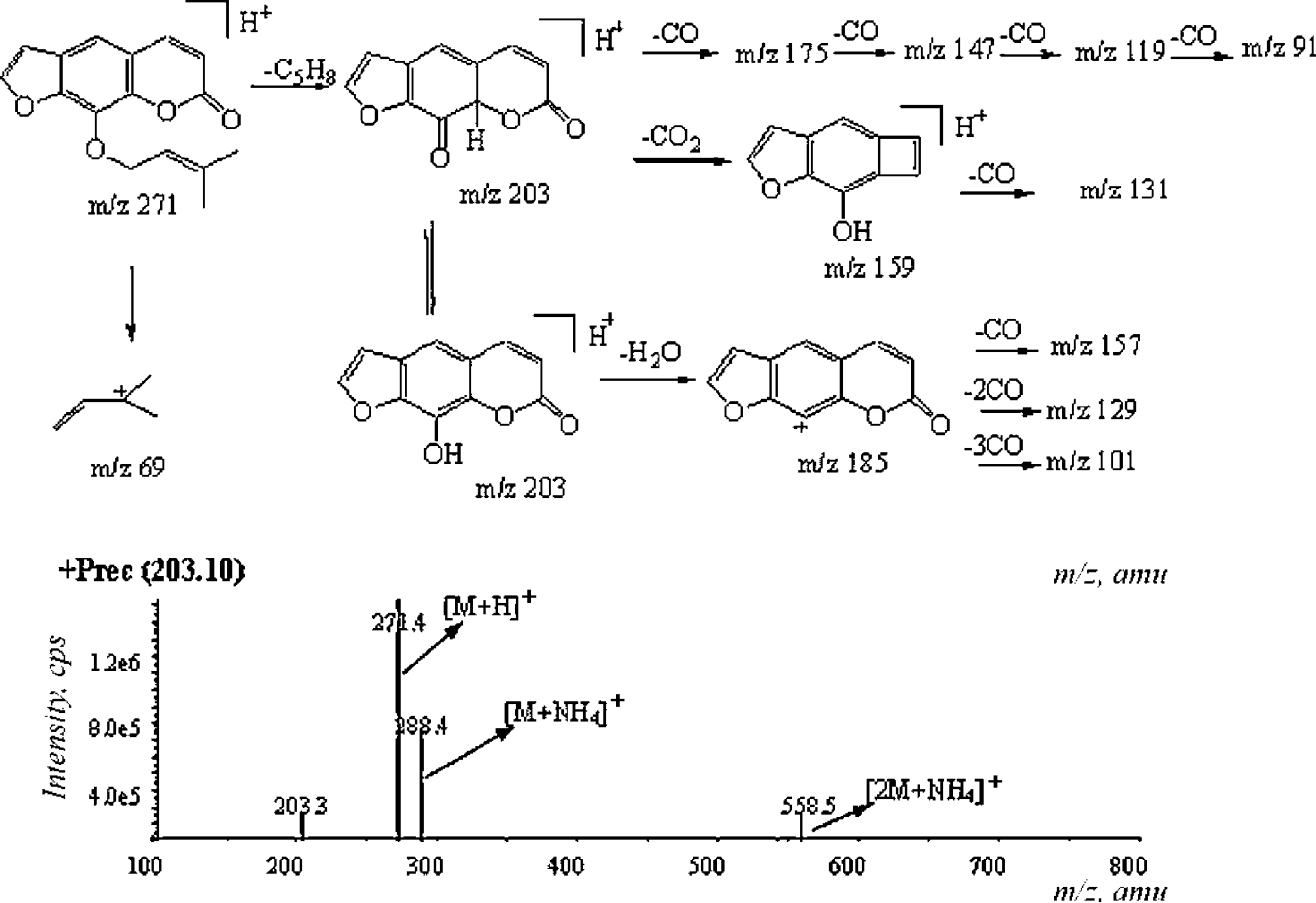
The mass spectral fragmentation pattern of imperatorin (Yang et al. 2010)

Characteristic values for the positive mass spectrometry ions [M+H]^+^ and [M+Na]^+^ of imperatorin are observed at *m*/*z* 271.1 and 293.0, respectively. Masses of the main fragment ions occur at *m*/*z* 203.1 and 147.2 (Liu et al. 2011). The mass spectral fragmentation pattern for imperatorin is presented in Fig. 4.

Nuclear magnetic resonance spectroscopy is used for characterization of funacoumarins and suitable solvents for furanocoumarins are dimethyl sulfoxide (DMSO-*d*_6_) and methanol (CD_3_OD) (Szewczyk and Bogucka-Kocka 2012). Typical ^1^H NMR data for imperatorin are as follows: ^1^H NMR (400 MHz, CDCl_3_) *δ*: 1.72, 1.74 (6H, s, 2 × CH_3_), 5.00 (2H, d, *J* = 7.2 Hz, H-1′), 5.61 (1H, m, H-2′), 6.37 (1H, d, *J* = 10 Hz, H-3), 6.82 (1H, d, *J* = 2.0 Hz, H-11), 7.36 (1H, s, H-5), 7.69 (1H, d, *J* = 2.0 Hz, H-12), and 7.77 (1H, d, *J* = 9.6 Hz, H-4) (Liu et al. 2004b).

## Biotechnological methods to produce imperatorin

In order to increase the concentration of imperatorin in raw plant material for enhanced extractive yield, studies of the biosynthesis of the active substance using biotechnological methods may be employed. The main goals of these studies are: to increase the content of desired target compound, to lower the level of undesirable compounds, and to induce novel compound production in a specific plant (Kirakosyan and Kaufman 2009). In the case of imperatorin production in plants, two methods were used: the culturing of suspension cells and the obtaining of hairy roots by *Agrobacterium* spp mediated transformation. Imperatorin was obtained from *A*. *dahurica* var. *formosana* by cell suspension cultures. The callus cells were treated with various nutritional factors and addition of the adsorbent Amberlite XAD-7 increased the yield of imperatorin 140-fold to 460 µg g^−1^ d.w (Mulabagal and Tsay 2004).

## Imperatorin as a future drug

Considering the variety of pharmacological properties displayed by imperatorin, it is clear that it has some prospects for development as a pharmaceutical formulation for potential assessment as a clinical entity. Presently, there are a few patents about the possible application of imperatorin as an antihypertensive, anti-osteoporosis, and heart protective drug summarized in Table 6.

**Table 6:** Examples of patents with imperatorin as the compound of interest

Patent title	Number	Country
Pharmaceutical application of imperatorin in treatment of hypertension complicated with myocardial hypertrophy and heart protection	CN102349891 (A)—2012-02-15	China (He 2012)
Furanocoumarin compounds with antihypertensive activity and preparation methods thereof	WO2011160597 (A1)—2011-12-29	China (He 2011)
Novel inexpensive and efficient process for isolation of imperatorin, a potent inducible nitric oxide synthase inhibitor and anti-inflammatory drug candidate from *Aegle marmelos* Correa	US20050220913 A1	USA (Ponnapalli Mangala 2005)

## Conclusions

Imperatorin is an important component of plants in the Apiaceae and Rutaceae families which have been used since antiquity in traditional medicine to treat a variety of ailments. Imperatorin alone may be useful in the treatment of disorders such as epilepsy, anxiety, depression, as an AChE inhibitor it has prospects in the therapy of Parkinson’s and Alzheimer’s diseases. Other significant biological properties have also been ascribed to imperatorin, including antibacterial, anticancer, antiostheoporotic, myorelaxant, anti-inflammatory, cardioprotective, hepatoprotective and inhibiting of HIV replication. It may also merit further biological and pharmacological assessment for the treatment of cardiovascular disorders, osteoporosis, AIDS, and cancer. This brief review accumulates the botanical, chemical, and biological information which suggests that imperatorin is worthy of additional, prioritized research studies as a prelude to selecting the next steps in a pathway from a plant to a medicinal agent.
